# Supplementary material for: Optical characterization of surface adlayers and their compositional demixing at the nanoscale
Source: Nat Commun. 2018 Apr 12;9:1435. doi: 10.1038/s41467-018-03820-w (PMC5897338; doi:10.1038/s41467-018-03820-w)
Supplement: Supplementary file 1 — Supplementary Information [file 41467_2018_3820_MOESM1_ESM.pdf]

Supplementary Information

Optical characterization of surface adlayers and their compositional demixing at the nanoscale

Limin Xiang[1], Michal Wojcik[1], Samuel J. Kenny[1], Rui Yan[1], Seonah Moon[1], Wan Li[1], Ke Xu*[1,2]

1. Department of Chemistry, University of California, Berkeley, California 94720, USA

2. Chan Zuckerberg Biohub, San Francisco, California 94158, USA

*Correspondence to: xuk@berkeley.edu

Table of contents

Supplementary Figure 1	Additional SR-PAINT images of TCE and chloroform adlayers	**3**
Supplementary Figure 2	The adlayers are highly stable under imaging conditions	**4**
Supplementary Figure 3	SR-PAINT results using Merocyanine 540	**5**
Supplementary Figure 4	Additional AFM images of TCE and chloroform adlayers	**6**
Supplementary Figure 5	AFM results under different modes	**7**
Supplementary Figure 6	Additional SR-PAINT images of tetrahydrothiophene adlayers	**8**
Supplementary Figure 7	Additional SR-PAINT images, and AFM images of ethyl acetate adlayers	**9**
Supplementary Figure 8	Additional SR-PAINT images, and AFM images of dichloromethane adlayers	**10**
Supplementary Figure 9	SR-PAINT images of nitroethane adlayers	**11**
Supplementary Figure 10	Additional SR-PAINT images, and AFM images of nitromethane adlayers	**12**
Supplementary Figure 11	Additional SR-PAINT images for ethyl acetoacetate adlayers	**13**
Supplementary Figure 12	Additional SR-PAINT results of the adlayers of a 1:3 TCE-chloroform mixture	**14**

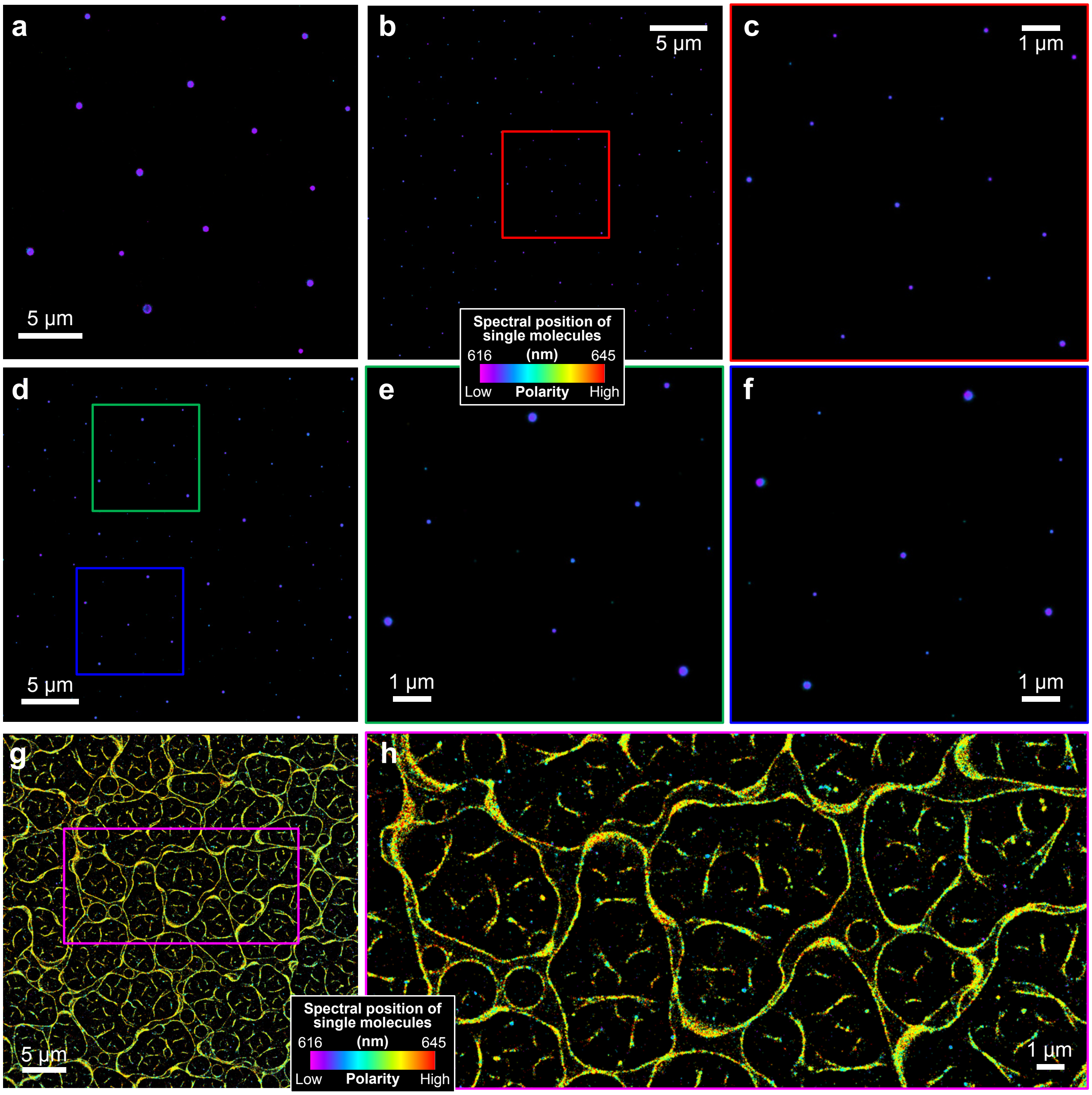

Supplementary Figure 1 | Additional SR-PAINT images of TCE and chloroform adlayers. a-f, TCE adlayers. **c** is a zoom-in of the box in **b**. **e** and **f** are zoom-ins of the boxes in **d**. **g-h,** chloroform adlayers. **h** is a zoom-in of the box in **g**.

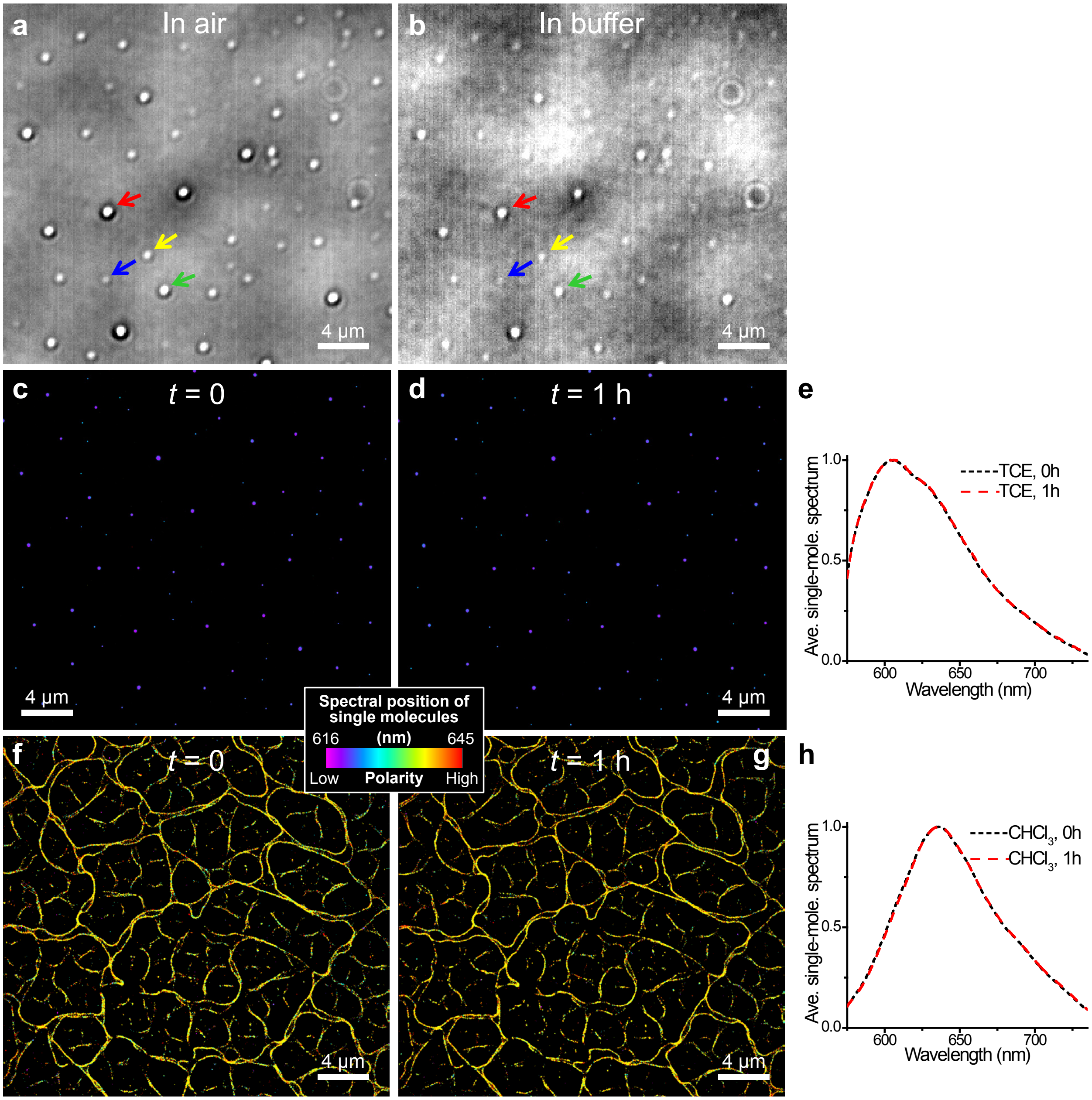

Supplementary Figure 2 | The adlayers are highly stable under imaging conditions. a, Differential interference contrast (DIC) microscopy of the as-prepared TCE adlayers on glass. Individual nanodroplets had similar apparent sizes of ~600 nm, limited by the DIC resolution, but showed different contrasts due to differences in their actual sizes. **b**, DIC result after adding the imaging buffer. A global decrease in image contrast occurred due to the reduced difference in index of refraction between water and TCE vs. air and TCE; meanwhile, the adlayer structure was unchanged. Arrows point to the same, representative droplets. **c-h**, SR-PAINT images and averaged single-molecule spectra for TCE (**c-e**) and chloroform (**f-h**) adlayers taken at 1 h apart, showing structural stability down to nanoscale details, as well as unvarying spectra (and hence polarity) over time.

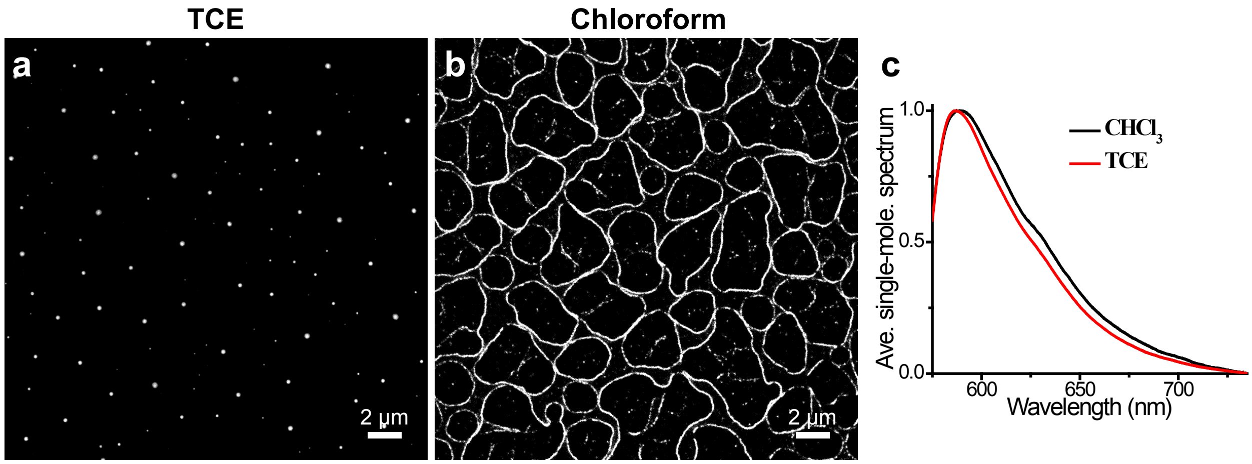

Supplementary Figure 3 | SR-PAINT results using Merocyanine 540. a-b, Merocyanine 540 PAINT images of TCE (**a**) and chloroform (**b**) adlayers. **c,** Averaged single-molecule spectra for the TCE (red curve) and chloroform (black curve) adlayers, showing minimal shifts between the two adlayers when compared to results of Nile Red (Figs. 1d and 4c).

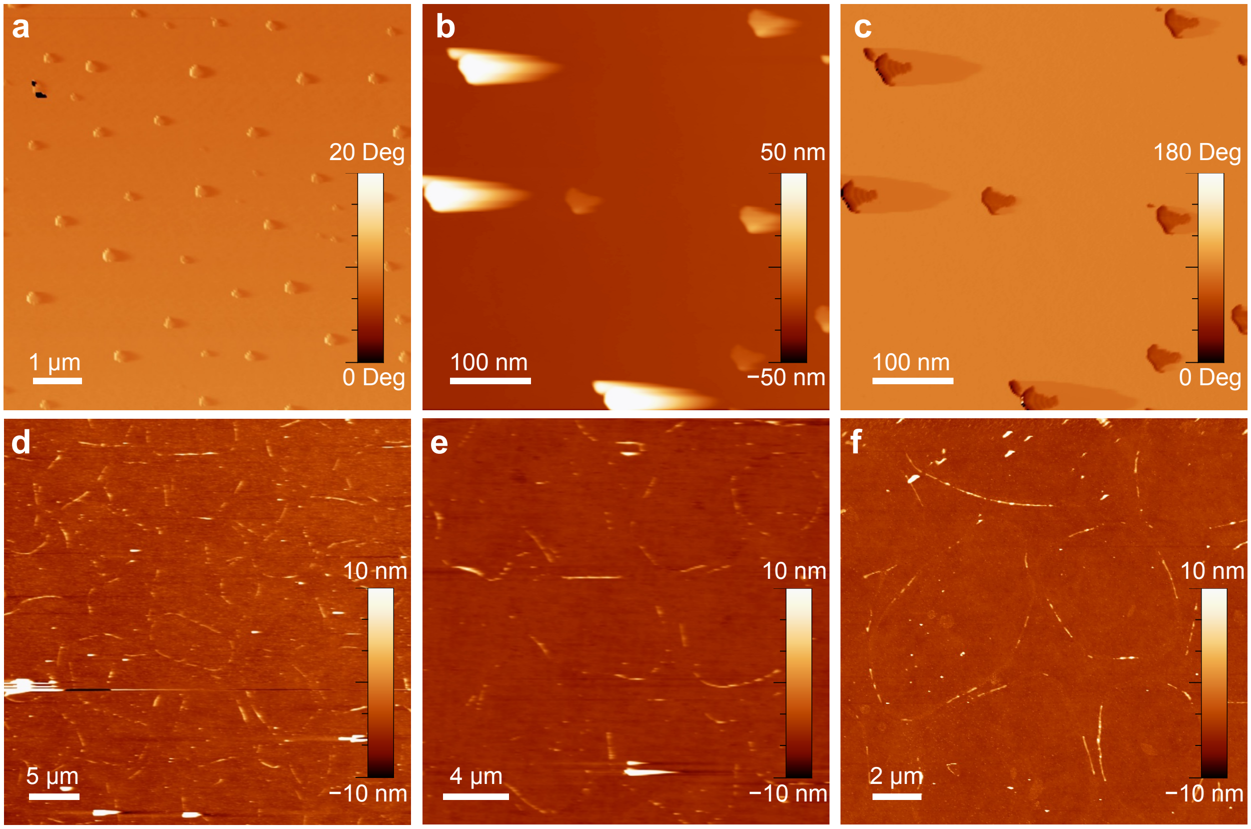

Supplementary Figure 4 | Additional AFM images of TCE and chloroform adlayers. a-c, TCE adlayers. **a,** AFM phase image corresponding to the height image in Fig. 2c. **b-c,** AFM height (**b**) and phase (**c**) images at high magnification. Droplets were severely deformed by the AFM tip. **d-f,** Chloroform adlayers. Height images at different magnifications all show incomplete circular network structures.

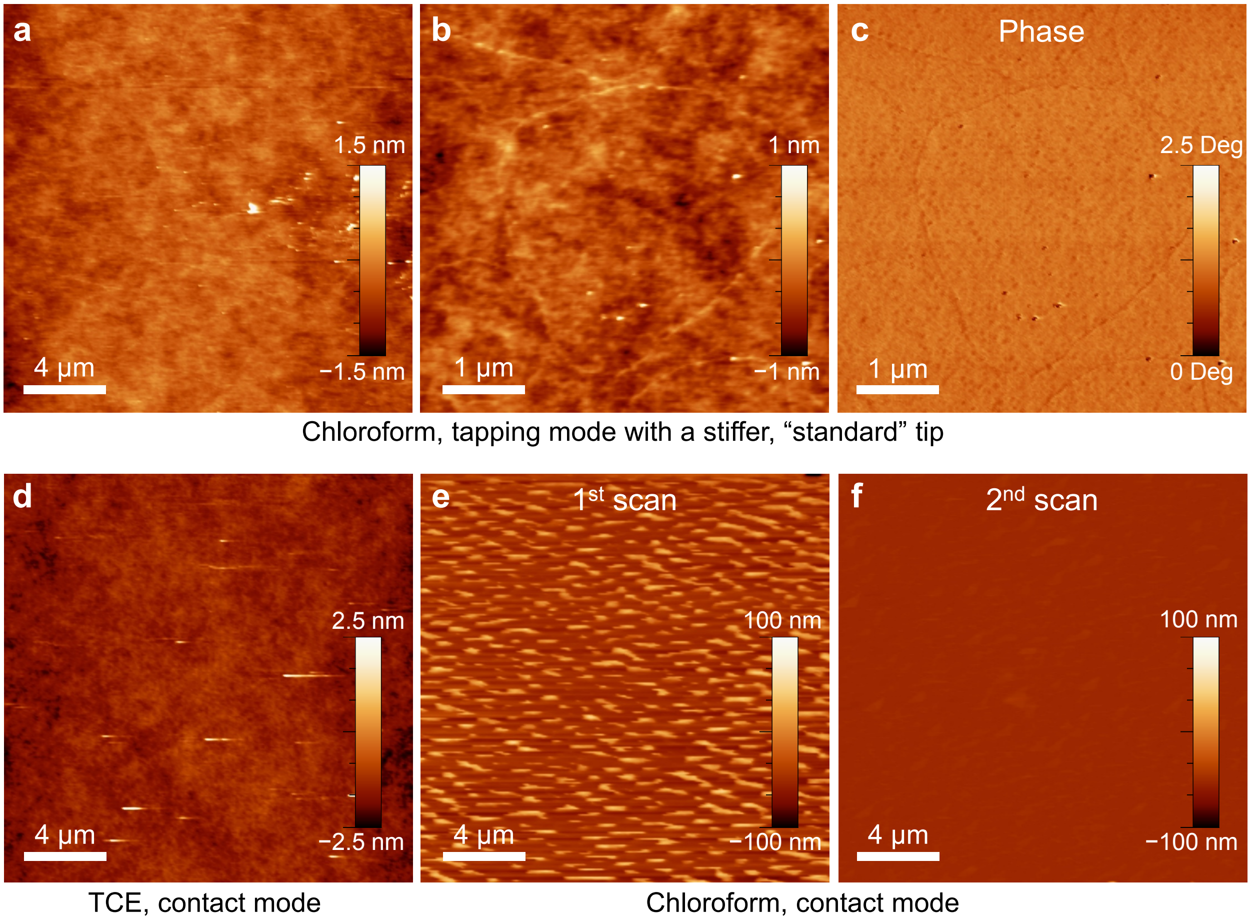

Supplementary Figure 5 | AFM results under different modes. a-c, Height (**a-b**) and phase (**c**) images of chloroform adlayers using a standard tapping-mode probe (nominal force constant: 48 $N \cdot m^{-1}$). Reduced adlayer contrast was found: The nano-line structures were not visible at moderate magnifications (**a**) and were barely visible at high magnifications (**b-c**). **d-f,** Contact-mode height images of TCE (**d**) and chloroform (**e-f**) adlayers. **e-f** are two consecutive scans of the same area. The AFM tip severely dragged the adlayers along, and so no adlayers were correctly visualized. (**f**) further indicates that the AFM tip removed the adlayers from the surface during scanning.

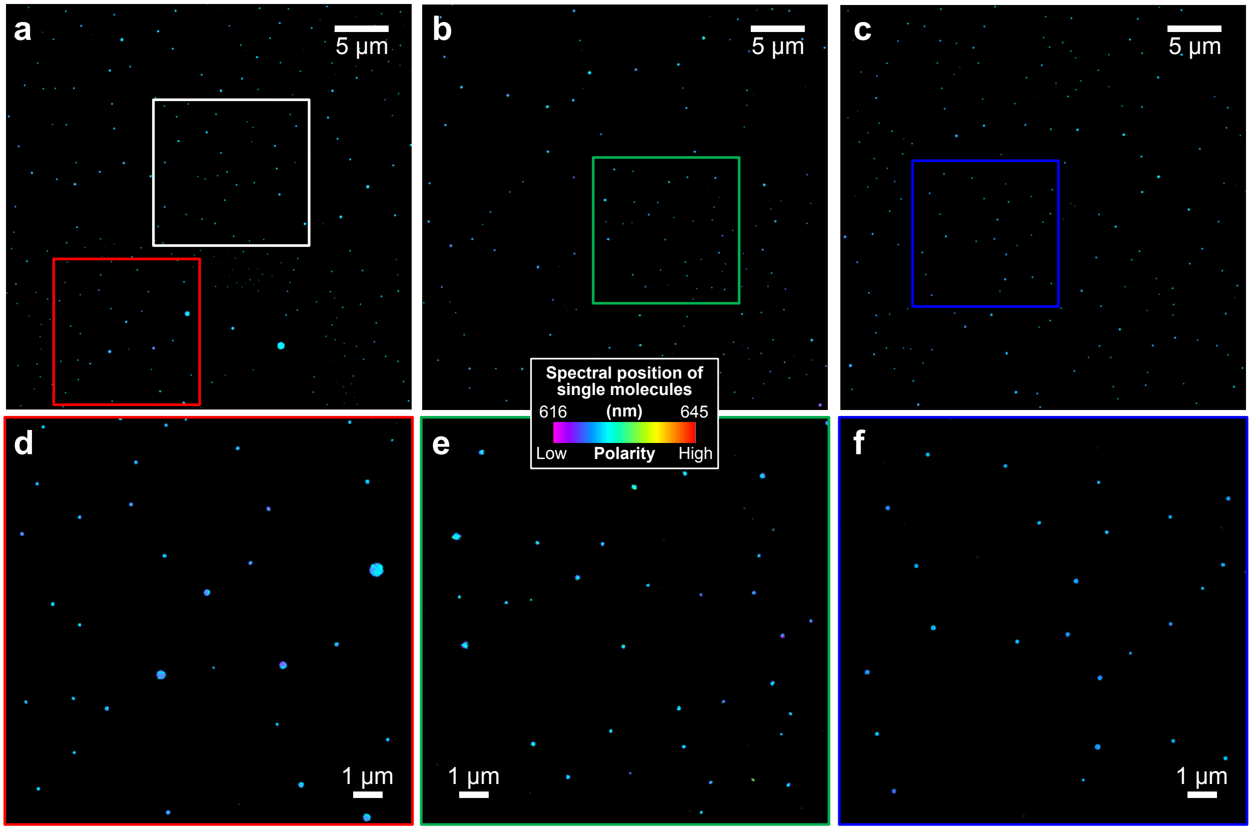

Supplementary Figure 6 | Additional SR-PAINT images of tetrahydrothiophene adlayers. White box in **a** corresponds to Fig. 3a. **d-f** are zoom-ins of the colored boxes in **a-c**, respectively.

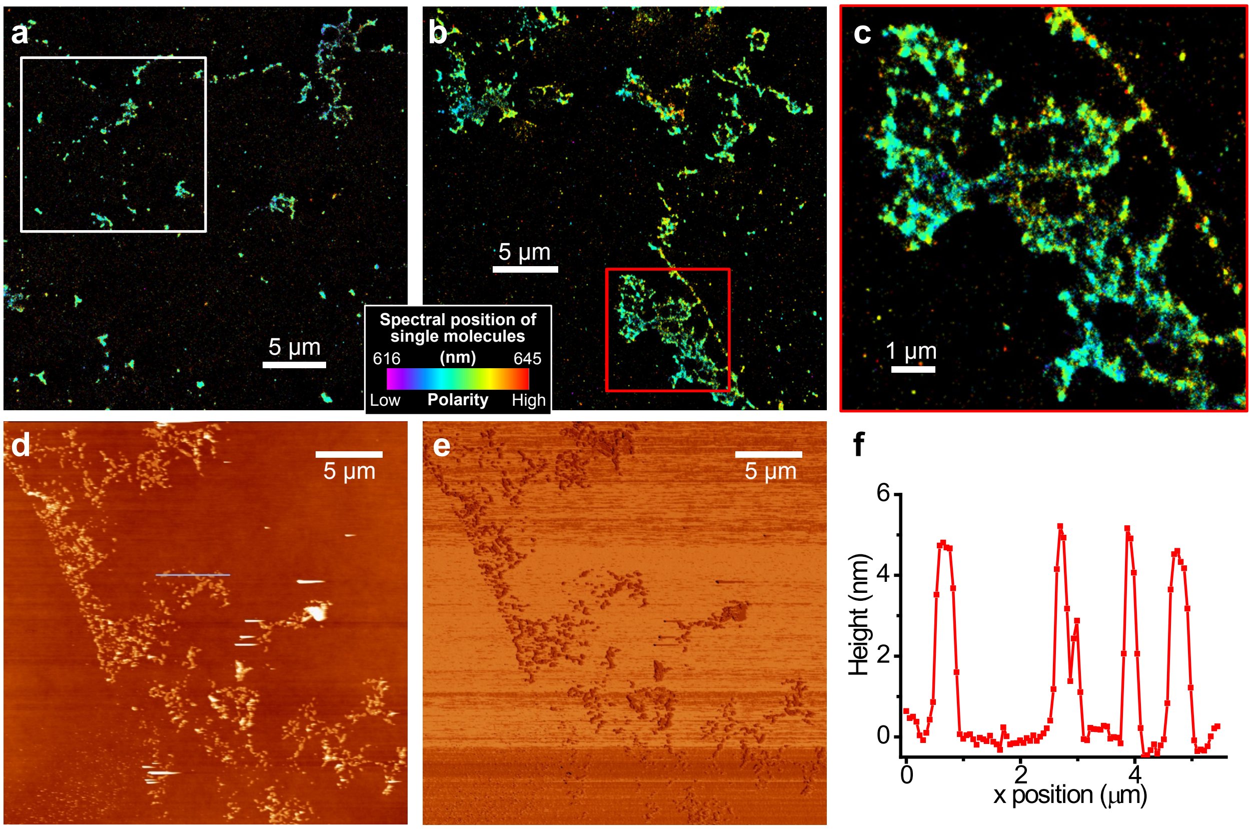

Supplementary Figure 7 | Additional SR-PAINT images, and AFM images of ethyl acetate adlayers. a-c, SR-PAINT images. White box in **a** corresponds to Fig. 3b. **c** is a zoom-in of the red box in **b**. **d-f,** AFM height (**d**) and phase (**e**) images, and a height profile (**f**) along the line marked in **d**.

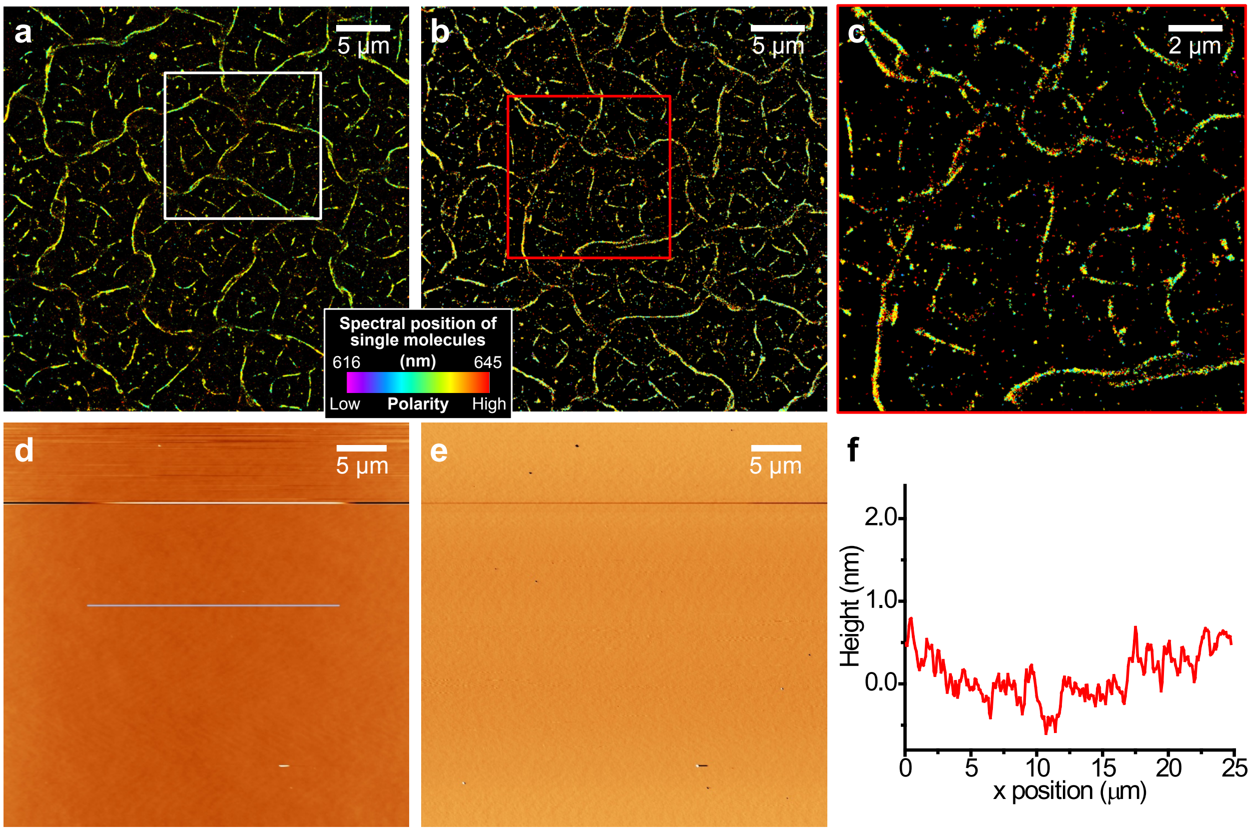

Supplementary Figure 8 | Additional SR-PAINT images, and AFM images of dichloromethane adlayers. a-c, SR-PAINT images. White box in **a** corresponds to Fig. 3c. **c** is a zoom-in of the red box in **b**. **d-f,** AFM height (**d**) and phase (**e**) images, and a height profile (**f**) along the line marked in **d**. No adlayer is detected in AFM, possibly due to the very low boiling point and high vapor pressure of dichloromethane.

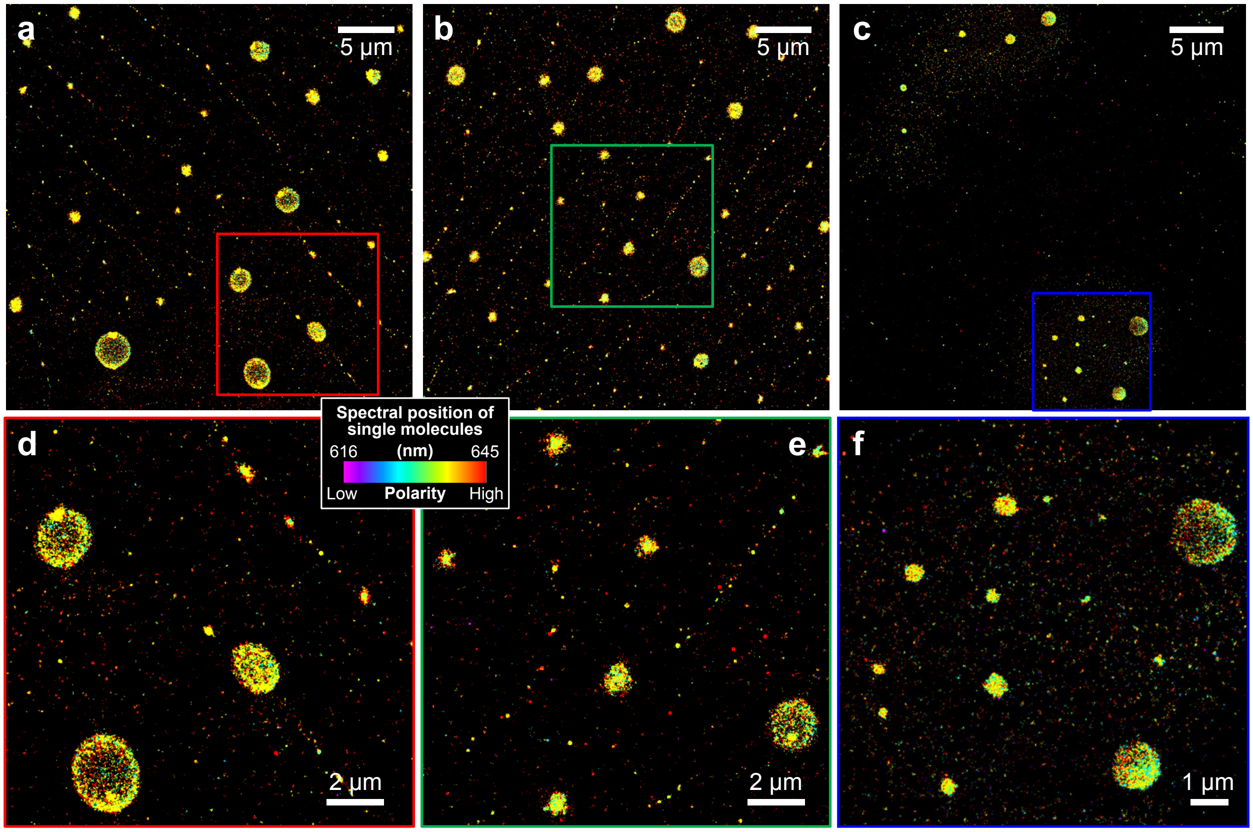

Supplementary Figure 9 | SR-PAINT images of nitroethane adlayers. d-f are zoom-ins of the colored boxes in **a-c**, respectively.

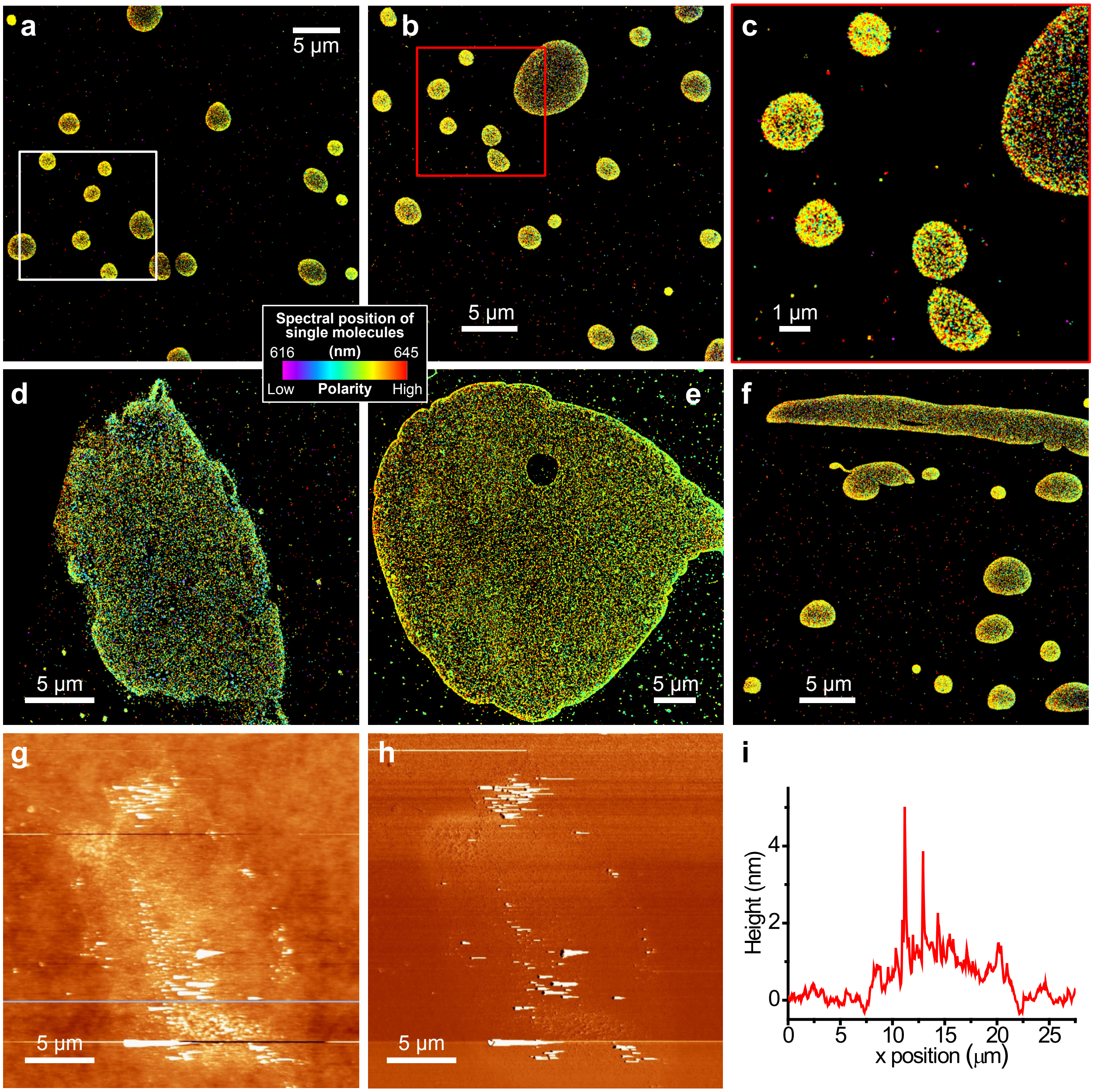

Supplementary Figure 10 | Additional SR-PAINT images, and AFM images of nitromethane adlayers. a-f, SR-PAINT images. White box in **a** corresponds to Fig. 3d. **c** is a zoom-in of the red box in **b**. **g-h,** Correlated AFM height (**g**) and phase (**h**) images for the same adlayer shown in (**d**). **i,** Height profile across the line in panel **g**.

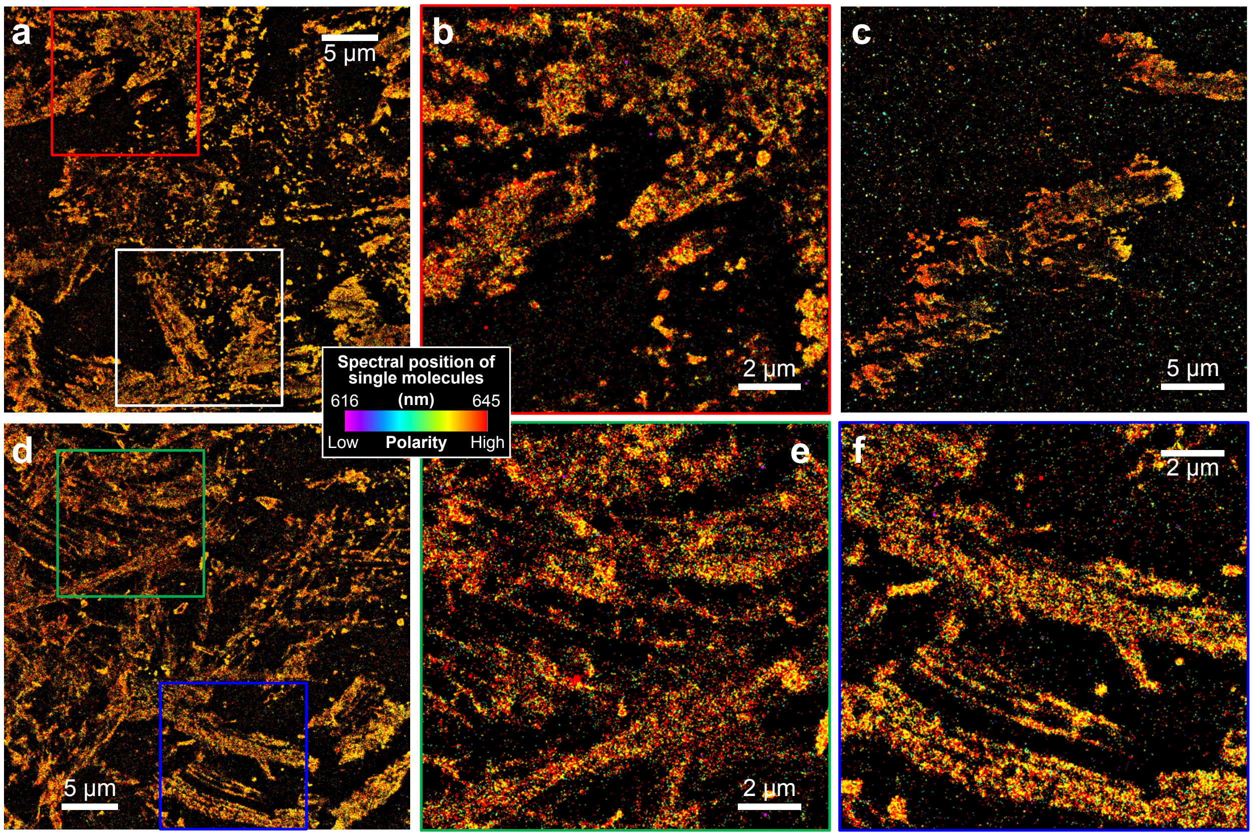

Supplementary Figure 11 │ Additional SR-PAINT images for ethyl acetoacetate adlayers. **a,c,d,** Results of three representative samples. White box in **a** corresponds to Fig. 3e. **b** is a zoom-in of the red box in **a**. **e** and **f** are zoom-ins of the green and blue boxes in **d**, respectively.

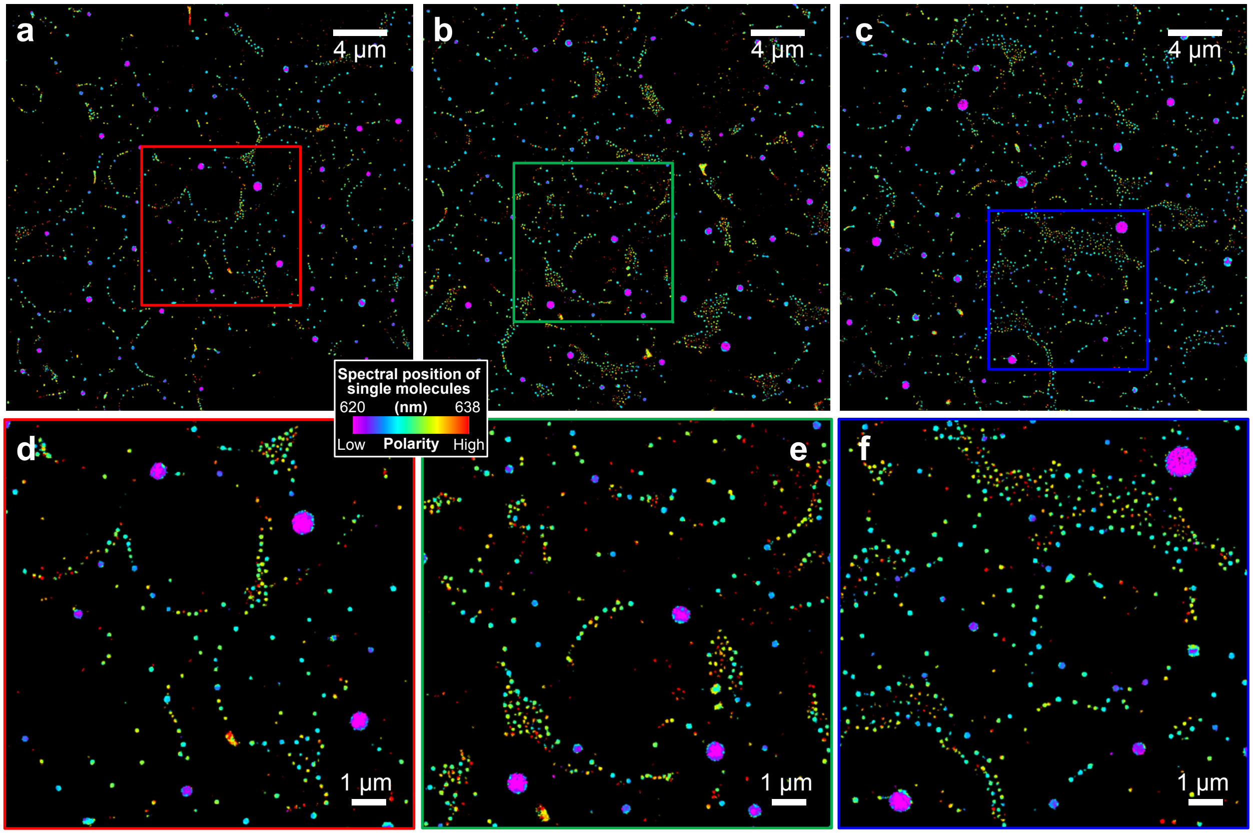

Supplementary Figure 12 | Additional SR-PAINT results of the adlayers of a 1:3 TCE-chloroform mixture. d-f are zoom-ins of the colored boxes in **a-c**, respectively.
